# Retraction: Heat Shock Protein 70 Expression Is Spatially Distributed in Human Placenta and Selectively Upregulated during Labor and Preeclampsia

**DOI:** 10.1371/journal.pone.0199804

**Published:** 2018-06-21

**Authors:** The PLOS ONE Editors

The *PLOS ONE* Editors retract this article [1] in light of concerns raised about Figure 2.

Similarities were noted between bands within the β-actin panel, which also appears to duplicate the β-actin panel in Figure 2 of [2].

In addition, lanes 1, 2 of the HSP 70 blot for labor samples (middle panel) appear similar to the bands shown in the β-actin blot (e.g. in lanes 2, 3).

The University of Glasgow investigated these concerns and recommended retraction owing to signs of data manipulation and falsification. In the course of the investigation, it was established that the original data underlying the figure panels in question are no longer available.

In light of these concerns, and in line with the institution’s communication, the *PLOS ONE* Editors retract this article, as the concerns raised call into question the integrity of the data and validity of the article’s results and conclusions.

AA agreed with the retraction. KH and FL did not respond.
