# Correction: Retraction: Heat Shock Protein 70 Expression Is Spatially Distributed in Human Placenta and Selectively Upregulated during Labor and Preeclampsia

**DOI:** 10.1371/journal.pone.0211126

**Published:** 2019-01-16

**Authors:** The PLOS ONE Editors

After this retraction notice [1] was posted, Akrem Abdulsid notified *PLOS ONE* that she had changed her position on the editorial decision in this case. The final paragraph of the retraction notice is hereby updated to the following: AA did not agree with the retraction. KH and FL did not respond.

## References

[1] The *PLOS ONE* Editors (2018) Retraction: Heat Shock Protein 70 Expression Is Spatially Distributed in Human Placenta and Selectively Upregulated during Labor and Preeclampsia. PLoS ONE 13(6): e0199804 10.1371/journal.pone.0199804 29928038 PMC6013219

